# Synthesis, Characterization, and Electrocatalytic Properties of PrMn_0.5_M_0.5_O_3_ (M = Cr, Fe, Co, Ni) Perovskites

**DOI:** 10.3390/ma18030717

**Published:** 2025-02-06

**Authors:** Besarta Cheliku Ramadani, Jeta Sela, Leon Stojanov, Sofija Popovska, Valentin Mirčeski, Miha Bukleski, Sandra Dimitrovska-Lazova, Arianit A. Reka, Slobotka Aleksovska

**Affiliations:** 1 Department of Chemistry, Faculty of Natural Sciences and Mathematics, University of Tetovo, Ilinden n.n., 1200 Tetovo, North Macedonia; besartaceliku@hotmail.com (B.C.R.); jeta.sela@unite.edu.mk (J.S.); 2 Institute of Chemistry, Faculty of Natural Sciences and Mathematics, Ss. Cyril and Methodius University, Arhimedova 5, 1000 Skopje, North Macedonia; leon_stojanov@yahoo.com (L.S.); sofija_popovska_1997@hotmail.com (S.P.); valentin@pmf.ukim.mk (V.M.); mihabukleski@pmf.ukim.mk (M.B.); sandra@pmf.ukim.mk (S.D.-L.); bote.aleksovska@gmail.com (S.A.); 3 Research Center for Environment and Materials, Macedonian Academy of Sciences and Arts, Bul. Krste Misirkov 2, 1000 Skopje, North Macedonia; 4 NanoAlb, Albanian Unit of Nanoscience and Nanotechnology, Academy of Sciences of Albania, Fan Noli Square, 1000 Tirana, Albania

**Keywords:** double manganese perovskites, XRPD, SEM, EDX, IR spectroscopy, cyclic voltammetry

## Abstract

In this paper, the synthesis, characterization, and investigation of electrocatalytic properties of perovskites of general formula PrMn_0.5_M_0.5_O_3_ (M = Cr, Fe, Co, Ni) are presented. The synthesis was conducted by the solution combustion method using glycine as a fuel. The perovskite with the formula PrMn_0.5_Fe_0.5_O_3_ was also synthesized by the sol–gel combustion method with citric acid as fuel. The obtained perovskites were investigated by X-ray powder diffraction (XRPD), scanning electron microscopy (SEM) with energy dispersive X-ray spectroscopy (EDX), infrared spectroscopy, and cyclic voltammetry. The XRPD patterns showed that the compounds are pure and isostructural within the series. The unit cell parameters of the compounds were determined within the *Pnma* space group, and several crystallochemical parameters were calculated and discussed. The recorded SEM images of the perovskites revealed a porous morphology, while the EDX analysis confirmed the 2:1:1 atomic percentage ratio of Pr:Mn:M. Within this investigation, the electrocatalytic properties of the obtained perovskites towards oxidation of OH^−^ ions and H_2_O_2_ oxidation in phosphate buffer were studied by cyclic voltammetry, using a paraffin-impregnated graphite electrode (PIGE) modified with microcrystals of the investigated perovskites. PrMn_0.5_Fe_0.5_O_3_ showed high electrocatalytic activity for OH^−^ oxidation, while both PrMn_0.5_Fe_0.5_O_3_ and PrMn_0.5_Co_0.5_O_3_ exhibited significant efficiency for H_2_O_2_ oxidation, with a distinct oxidation peak with a peak potential of 0.6 V.

## 1. Introduction

Perovskite-type compounds (ABX_3_) are among the most studied materials in solid-state chemistry and physics since they have emerged as materials with various applications due to their flexible composition and structure [1,2,3,4,5]. Small changes in their elemental composition can affect their structure and, consequently, their physicochemical properties, leading to a number of fundamentally interesting and important electrical, magnetic, catalytic, etc., properties [6,7], some of them being unique, such as multiferroicity, colossal magnetoresistance, etc. [8,9,10,11]. Recently, lead halide perovskites with organic cations in the A position attracted special attention due to their applications for efficient solar cell production [11]. Alternatives for replacement of highly polluting lead halide perovskites are the nontoxic and chemically stable layered bismuth halide perovskites of general formula A_3_Bi_2_X_9_ (A = K^+^ Rb^+^, Cs^+^, or methylammonium MA^+^, and X = I^−^, Br^−^, or Cl^−^) [12]. These perovskites are promising materials for the photocatalytic reduction of CO_2_ to different fuels, such as CH_3_OH, CH_4_, C_2_H_5_OH, etc., and thus decrease the CO_2_ concentration in the atmosphere and produce solar fuels to mitigate the dependence on highly polluting fossil fuels.

Regarding oxide perovskites (ABO_3_), the most common way to tailor their properties is partial substitution in the A position, B position, or both, leading to the formation of double or quadrupole perovskites [1,13,14,15]. Most of the applicable properties of perovskites typically depend on B-cations, particularly when combined with a rare-earth element in the A position [14,15]. The combination of two transition metals in the B position can enhance certain properties or introduce new ones. Thus, much attention has been given to A_2_BB′O_6_ perovskites, where A is a rare-earth element and BBʹ are transition elements [14,15,16]. The B-site substitution can occur in varying amounts, but the perovskites with AB_0.5_B′_0.5_O_3_ stoichiometry are the most investigated [1,13,14,15,16]. Different oxidation state combinations are possible in B-cations, and their arrangement may be ordered or disordered [1,13,14,15,16,17,18]. These double perovskites have been demonstrated to be effective electrocatalysts for the oxygen evolution reaction (OER) [19,20,21,22] and the oxygen reduction reaction (ORR) [23] in both acidic and basic media.

Among the diverse perovskites, those oxide perovskites containing manganese in the B position and certain lanthanides in the A position stand out due to the display of various magnetic and electric phenomena, as well as their catalytic properties [1,9,24,25]. This is attributed to the presence of manganese in both Mn^3+^ and Mn^4+^ states within the perovskite structure, leading to the formation of perovskites with Ln or Mn deficiency or oxygen excess [1,9,24]. Another important factor influencing the structure and properties of Mn-perovskites is the distortion of Mn^3+^O_6_ octahedron due to the Jahn–Teller effect of high-spin Mn^3+^ ions (d^4^, t_2g_^3^e_g_^1^) [1,9,24,25]. The mixed valent state (Mn^3+^/Mn^4+^) is also present in alkaline earth-doped perovskites Ln_1−*x*_A*_x_*MnO_3_ in order to maintain charge balance when the alkaline earth cation substitutes Ln^3+^. This specific type of perovskite is particularly noteworthy for displaying colossal magnetic resistance affected by the double exchange mechanism, which is manifested by the hopping of an electron from high-spin Mn^3+^ to Mn^4+^ cation via the intermediate oxygen ion [1,9,24,25,26].

Considerable attention has also been given to double Mn-based perovskites, REMn_0.5_M_0.5_O_3_ (RE_2_MnMO_6_, M is a transition metal), due to their potential as multifunctional materials [14,15,16,24,25,27]. Generally, the RE_2_MnMO_6_ perovskites can exist in an ordered state, usually adopting the monoclinic structure, or in a disordered state with an orthorhombic structure, which is in most cases dependent on the applied synthesis method [1,24,27,28]. The most investigated perovskites of this type are RE_2_MnNiO_3_. Recently, Baral et al. summarized the investigation on these compounds in a review article [29], concluding that there is still much work to be carried out in investigating RE_2_MnMO_6_. The focus of our research is on Pr_2_MnMO_6_ perovskites. Zhang et al. [30] investigated the magnetic properties of Pr_2_MnNiO_6_ and Pr_2_MnCoO_6_ perovskites synthesized by a classical solid-state reaction and found that both crystallize in the monoclinic *P2*_1_/*n* structure. Recently, Elhamel et al. presented a comprehensive study on RE_2_MnNiO_6_ (RE = Pr, Gd, Er) as nanoceramics, obtained by the sol–gel method [31]. Rawat et al. [32] investigated the structural and magnetic properties of the nanocrystalline Pr_2_MnCoO_6_ perovskite obtained by the sol–gel method, which crystalizes in the *Pbnm* orthorhombic space group. The citrate–gel method was used by Aswathi et al. [33] for the synthesis of Pr_2_MnFeO_6_ with an orthorhombic structure (*Pbnm*). The same method was applied by Muddelwar et al. for the synthesis of the Pr_2_MnCrO_6_ perovskite [34], resulting in a monoclinic (*P2*_1_/*n*) structure.

Given the current focus on research of Pr_2_MnMO_6_ (M = Fe, Co, Cr, Ni), here we present the solution combustion synthesis of these compounds and the resulting structural features related to it. In the past few decades, solution combustion synthesis (SCS) became one of the most applied methods for ceramic and composite materials synthesis, including the perovskites. This method is simple, fast, energy efficient, and low cost. The final products are of great phase purity, with small particle size (usually nanosized) and good sintering properties. By controlling the procedure conditions and parameters (such as the type and amount of the fuel), it is possible to tailor the morphology and the properties of the products and thus their application [35]. Although these compounds are investigated from different points of view, there are no data on their electrocatalytic properties. Therefore, here we also present the investigation of their electrocatalytic properties performed by cyclic voltammetry.

## 2. Experimental Section

### 2.1. Materials

All the following starting materials were purchased from Sigma-Aldrich, were of analytical grade, and were used without further purification treatment: praseodymium(III) nitrate pentahydrate (99.9%), glycine (≥98.5%), citric acid anhydrous (≥99.5%), nitric acid (65%), cobalt(II) nitrate hexahydrate (≥98%), manganese(II) nitrate tetrahydrate (≥98.5%), nickel(II) nitrate hexahydrate (≥98.5%), and chromium(III) nitrate nonahydrate (≥98%).

### 2.2. Syntheses of Perovskites

The synthesis of the proposed perovskite series with the general formula PrMn_0.5_M_0.5_O_3_ (M = Fe, Co, Cr, Ni) was performed using the solution combustion method with glycine as fuel. Stoichiometric amounts of nitrates of the constituent metals were dissolved in a small amount of distilled water and mixed together (Supplementary Materials File/Tables S1–S4)). The fuel quantity was calculated to achieve a fuel-to-oxidizer ratio of 1 (φ = 1), aiming to attain the maximum temperature during combustion. The fuel was also dissolved in a small amount of distilled water and added to the solution of metal nitrates. The mixture of nitrates and glycine was heated at 70–80 °C until most of the water evaporated, forming a gel. Subsequently, the temperature was elevated to approximately 300 °C to initiate the combustion reaction, resulting in a black, sponge-like product. This product underwent further annealing at 800 °C for 8 h, followed by an additional 4 h at 900 °C. The schematic representation of the synthesis is presented in (Graphical Abstract).

In addition to the previously mentioned glycine-based method, the synthesis of Pr_2_MnFeO_6_ was also conducted using a modified sol–gel approach. In this method, citric acid was utilized as a complexing chelating agent and as a fuel. The synthesis initiates by dissolving stoichiometric amounts of praseodymium, manganese, and iron nitrates in water and heating the solution on a magnetic stirrer up to 70–80 °C. Upon reaching this temperature, the dissolved citric acid was added. In this synthesis, it is crucial to control the solution’s pH, maintaining it within the range of 5–6, as this ensures the optimal pH for the complexation reaction of iron with citric acid. Since the solution becomes acidic after adding citric acid, the desired pH was achieved by continuously adding 25 % ammonium hydroxide dropwise. Simultaneously, the temperature must be kept below 90 °C. With gradual heating, the solution slowly evaporates, resulting in the formation of a light brown gel. Once the gel was obtained, the beaker was transferred from the magnetic stirrer to a hot plate, where the gel undergoes complete combustion, forming a black, spongy precursor. The obtained precursor was then annealed at a temperature of 800 °C for 8 h, followed by an additional 4 h at 900 °C.

### 2.3. Powder X-Ray Diffraction

The crystallographic characteristics and the purity of the obtained perovskites were studied by X-ray powder diffraction (XRPD). The XRPD patterns were recorded on a Rigaku Ultima IV powder X-ray diffractometer equipped with Cu K_α_ radiation and a high-speed Det X detector. The diffractograms were recorded at room temperature within the 2*θ* range of 10–100° at a scanning rate of 1°/min.

### 2.4. Scanning Electron Microscopy

The morphology of the samples was studied by scanning electron microscopy (SEM) with energy-dispersive X-ray spectroscopy (EDX). Images and elemental composition were acquired using a FEI Quanta 3D FEG dual beam microscope using an accelerating voltage of 200 V to 30 kV for electron beam imaging and 5 to 30 kV for ion beam imaging. The samples were placed on a graphite strip and were adhered to gold coating before imaging with the microscope. Recordings were made using an in-lens and/or Everhardt–Thornley secondary electron detector.

### 2.5. Infrared Spectroscopy

The IR spectra were recorded in the far IR region starting from 700 to 30 cm^−1^ on a PerkinElmer Spectrum 3 spectrometer in a transmission mode. The samples were prepared by making a suspension of the solid material in nujol and were recorded between polyethylene plates. Both the background and all transmission spectra were recorded using 128 scans with a resolution of 2 cm^−1^ at room temperature. The CO_2_/H_2_O compensation software was used to reduce the bands originating from CO_2_ and atmospheric moisture. The recorded spectra were analyzed using the Spectrum IR 10 and Origin 9.0 software.

### 2.6. Cyclic Voltammetry

The electrocatalytic activity of the PrMn_0.5_M_0.5_O_3_ perovskites towards oxidation of OH^−^ ions and H_2_O_2_ was investigated by cyclic voltammetry. For this purpose, small amounts of perovskite microparticles were deposited by abrasion on a paraffin-impregnated graphite electrode (PIGE), which was used as a working electrode. Such a modified PIGE electrode was connected to the electrochemical cell consisting of a graphite electrode as a counter electrode and saturated Ag/AgCl (KCl; − 3 mol/dm^3^) as a reference electrode. Therefore, all potentials in the electrochemical data are reported relative to this reference electrode. All voltammograms were recorded with μAUTOLAB, model III potentiostat/galvanostat in the potential range of −1.5 V to +1.6 V, with a scan rate (*v*) of 50 mV/s and a potential step (∆*E*) of 1 mV, starting from OCP to the positive potential direction. By convention, in the CVs, a positive current represents oxidation processes, while a negative current represents reduction processes. These measurements were conducted at room temperature, and each individual voltammogram comprised three consecutive cycles. The activity of perovskite-modified electrodes toward the oxidation of OH^−^ ions (oxygen evolution reaction, OER) was studied in a 0.1 mol/dm^3^ KOH solution with 0.5 mol/dm^3^ KNO_3_ used as a supporting electrolyte. Additionally, the oxidation of H_2_O_2_ using a perovskite-modified electrode was studied in a 10^−2^ mol/dm^3^ H_2_O_2_ solution with 0.1 mol/dm^3^ phosphate buffer (pH = 7.35) used as a supporting electrolyte.

## 3. Results and Discussion

### 3.1. XRPD and Crystallochemical Calculations

**Figure 1:**
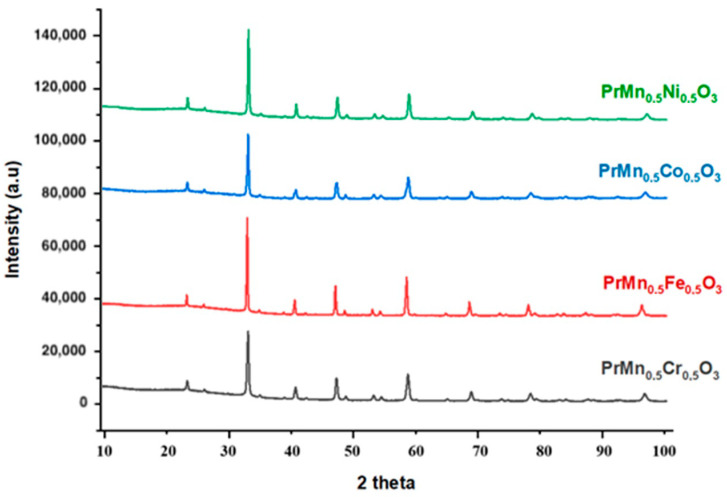
X-ray powder diffraction patterns of PrMn_0.5_M_0.5_O_3_ (M = Cr, Fe, Co, Ni) perovskites.

The recorded XRPD patterns of PrMn_0.5_M_0.5_O_3_ (or Pr_2_MnMO_6_) perovskites (M = Cr, Fe, Co, Ni) indicated that the studied compounds can be successfully synthesized using the solution combustion method with glycine as fuel upon annealing at 900 °C (Figure 1). The characterization of the synthesized compounds and the assessment of their purity were accomplished by comparing the recorded diffractograms with the patterns of simple perovskites [36] and the oxides of constituent metals. The compounds are pure, and according to their XRPD patterns, these perovskites are isostructural among themselves.

**Figure 2:**
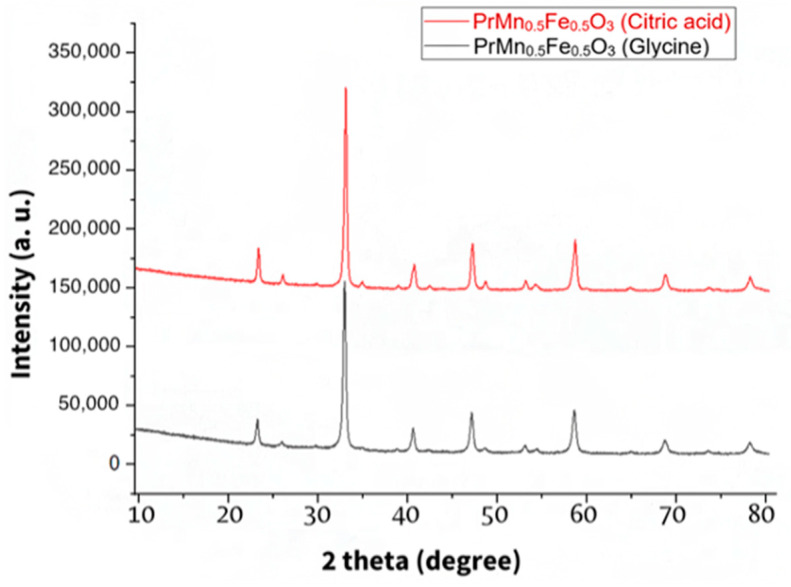
The XRPD patterns of the PrMn_0.5_Fe_0.5_O_3_ perovskite, obtained by two different synthetic procedures.

As previously detailed in the Experimental Section, the perovskite PrMn_0.5_Fe_0.5_O_3_ was synthesized using two distinct methods: the solution combustion method and the sol–gel combustion method. X-ray images confirm that the compounds obtained through both methods yield the same compound (Figure 2). This suggests that both methodologies are applicable for the synthesis of these double perovskites.

The diffractograms of the obtained compounds exhibit a similar peak arrangement as in the corresponding simple orthorhombic (*Pnma*) perovskites, with slight shifts in 2*θ*. Therefore, it can be assumed that the synthesized perovskites are orthorhombic as well and crystallize in the same space group. Also, according to Baral et al. [29], when the glycine solution combustion method is applied, the orthorhombic structure is usually obtained. Thus, the unit cell parameters were determined within the *Pnma* space group. The unit cell parameters (*a*, *b*, and *c*) were used to calculate several distortion indices (Table 1). Spontaneous strain (*s*) is an indicator of the deviation of the perovskite structure from cubic symmetry and is also used as a measure of the amount of deformation in octahedrons [37]. The cell distortion parameter (*d*) estimates the deviation of the perovskite from the ideal cubic structure [38] and the orthorhombic distortion (*dist*_orth_) [39], which is defined as a standard deviation divided by the average of the lattice parameters. These crystallochemical parameters and the tolerance factor (*t*) were calculated by the following equations:

(1) $$s=\frac{2\left(a-c\right)}{\left(a+c\right)}$$

(2) $$d=\frac{{\left(a/\sqrt{2}-a_{p}\right)}^{2}+{\left(c/\sqrt{2}-a_{p}\right)}^{2}+{\left(b/2-a_{p}\right)}^{2}}{{3a}_{p}^{2}}\times 10^{4}$$

where *a*_p_ is the aristotype cubic lattice parameter, which was calculated by the equation

(3) $$a_{p}=\frac{a/\sqrt{2}+c/\sqrt{2}+b/2}{3}$$

(4) $${\mathrm{dist}}_{\mathrm{orth}}=\frac{\sqrt{\sum {\left(a_{i}-\overset{-}{a}\right)}^{2}}}{\overset{-}{a}}$$

(5) $$t=\frac{r\left(\mathrm{Pr}\right)+r\left(O\right)}{\sqrt{2}\cdot \left[(r\left(\mathrm{Mn}\right)+r\left(M\right))/2+r(O)\right]}$$

The values of the ionic radii of the constituents in (5) correspond to the effective ionic radii taken from Shannon [40]. The cations in the B position are in the +3 oxidation state and six-coordinated, while the effective ionic radii of Pr^3^⁺ correspond to a coordination number of nine. Since oxygen is a weak field ligand, the values of the effective ionic radii for Mn^3^⁺, Fe^3^⁺, and Ni^3^⁺ correspond to their high spin states (t_2g_^3^e_g_^1^, t_2g_^3^e_g_^2^, and t_2g_^5^e_g_^2^, respectively), but for Co^3^⁺, it corresponds to its low spin state (t_2g_^6^), as it is in most of the other Co-containing perovskites.

The calculated values of the tolerance factors hover around 0.9, suggesting that the compounds exhibit a distorted perovskite structure. The unit cell parameters of the investigated compounds are of similar values, and for each compound, *a* > *c*, which is characteristic of the transition from a cubic to orthorhombic structure (*Pnma*) due to the tilting of the octahedra. The volumes of the unit cell increase in accordance with the ionic radii of M^3+^ [*r*(Co^3+^) l.s. < *r*(Ni^3+^) < *r*(Cr^3+^) < *r*(Fe^3+^)], as well as the values of the pseudocubic cell, *a*_p_. The comparison of *a, b√2*, and *c* may be used as an indicator of additional structural distortions. In the case when the relation between the parameters is *a* > *b*√2 > *c*, it is expected that octahedral tilting is primarily the only distortional mechanism. However, this relationship is fulfilled only for PrMn_0.5_Fe_0.5_O_3_.

To evaluate the overall distortion of the perovskite structure in the series, the cell distortion (*d*), the orthorhombic distortion (*dist*_orth_), and spontaneous strain (*s*) were calculated. For all of the investigated compounds, these values are small, indicating slightly distorted and stable structures. It is evident that the cell distortion, the orthorhombic distortion, and the internal strain are significantly low in PrMn_0.5_Ni_0.5_O_3_. However, it should be pointed out that the *a* and *c* lattice parameters are of very close values, meaning that the structure is orthorhombic with a pseudotetragonal unit cell. The values for internal strain in the other three perovskites are very close, indicating similar stability. The obtained values for cell distortion and for orthorhombic distortion both change in the same manner within this series: PrMn_0.5_Ni_0.5_O_3_ < PrMn_0.5_Fe_0.5_O_3_ < PrMn_0.5_Cr_0.5_O_3_ < PrMn_0.5_Co_0.5_O_3._

### 3.2. Morphology and Elemental Composition

**Figure 3:**
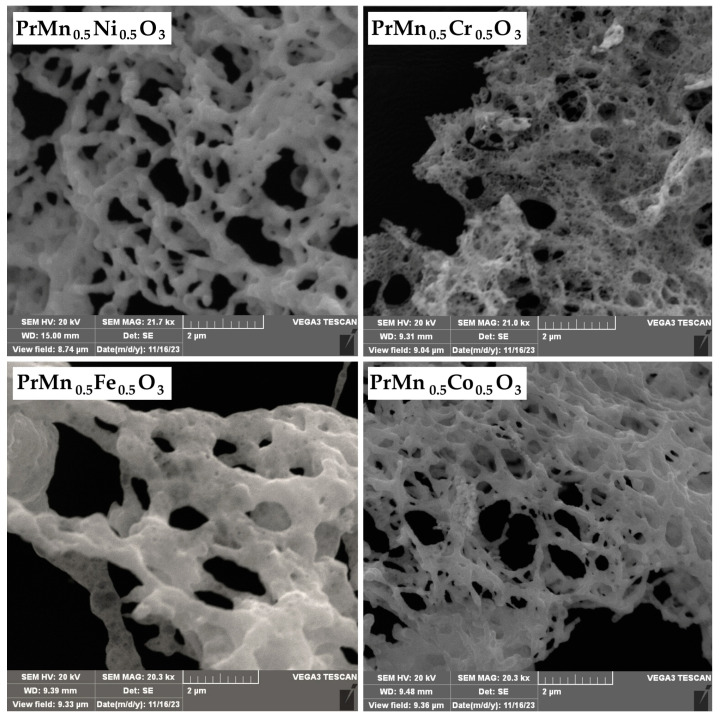
SEM images of PrMn_0.5_M_0.5_O_3_ (M = Ni, Co, Cr, Fe).

To assess the impact of the applied synthesis method on the morphology and dimensions of the particles, SEM images of PrMn_0.5_M_0.5_O_3_ (M = Ni, Co, Cr, Fe) were recorded. Within this series, the compounds exhibit a consistent polycrystalline porous morphology characterized by dense grains, a characteristic feature of perovskites obtained through the solution combustion method. The particles predominantly display uniform size and aggregate to form an open–pore network structure (Figure 3).

EDX analysis was conducted in order to validate the formation of the perovskites and to confirm the stoichiometry. Various regions were selectively examined during the EDX measurement, and the corresponding peaks are illustrated in the EDX data (Table 2). Table 2 shows the atomic percentages of the metals. It can be observed that the Pr:Mn:M ratio of the atomic percentages in all cases is approximately 2:1:1.

### 3.3. Infrared Spectra of the Obtained Perovskites

**Figure 4:**
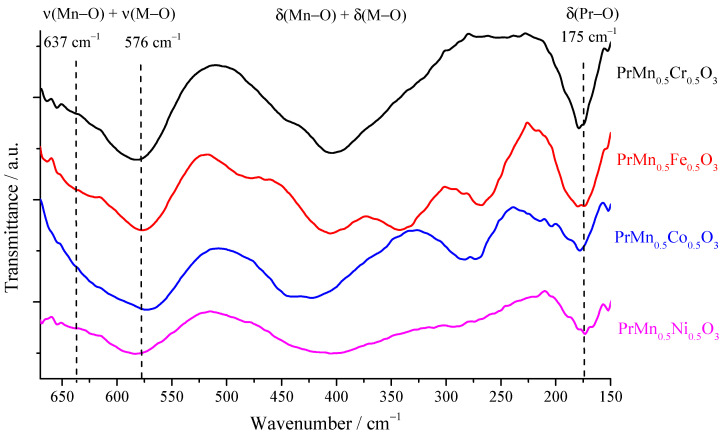
Infrared spectra of the PrMn_0.5_M_0.5_O_3_ (M = Ni, Co, Cr, Fe) series.

The recorded IR spectra in the far IR region of the synthesized perovskites are shown in Figure 4. In these spectra, the bands originating from the metal–oxygen stretching vibrations are in the region from 650 to 530 cm^−1^. In this region, the bands from the Mn–O and the M–O stretching vibrations overlap, i.e., the band from the M–O vibration is observed as a shoulder to the more intense band from the Mn–O vibration. On the other hand, the bands originating from the banding Pr–O vibrations appear at about 175 cm^−1^. These two sets of bands appear at about the same wavenumbers in the IR spectra of the investigated perovskites. This means that the substitution of the metal in the B position does not influence the site symmetry of praseodymium ions in the structure, nor its coordination. The same can be said for the position of the Mn atoms in the BO_6_ octahedra. This is expected and understandable since all the radii of the incorporated atoms (ions) are in close proximity. These results can lead to the conclusion that the compounds in this series are isostructural.

On the other hand, in the region from 520 to 270 cm^−1^, belonging to the bands originating from the Mn–O and the M–O banding vibrations, there are significant differences in the spectra of the investigated perovskites. These differences can be due to the different bond lengths between the metal in the B position and the oxygen atoms constituting the octahedra. This is also reflected in the band appearing as a shoulder to the band below 600 cm^−1^.

### 3.4. Electrocatalytic Properties

The electrocatalytic properties of a perovskite with the general formula PrMn_0.5_M_0.5_O_3_ (M = Ni, Co, Cr, Fe) in KNO_3_ (0.5 mol/dm^3^) were investigated by cyclic voltammetry.

**Figure 5:**
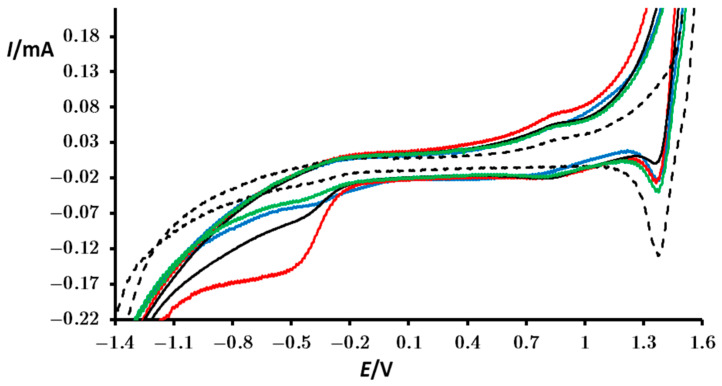
Cyclic voltammograms of a bare PIGE electrode (black dotted line) and PIGE modified with particles of PrMn_0.5_Ni_0.5_O_3_ (black), PrMn_0.5_Fe_0.5_O_3_ (green), PrMn_0.5_Cr_0.5_O_3_ (blue), and PrMn_0.5_Co_0.5_O_3_ (red) recorded in contact with a 0.5 mol/dm^3^ KNO_3_ used as a supporting electrolyte. These measurements correspond to a third consecutive scan. The potential was initially scanned from OCP in the positive direction. Additional conditions are specified in the Experimental Section.

For comparison, the cyclic voltammogram of the blank is also depicted (Figure 5—black dotted line). The oxidation current in the cyclic voltammogram of the blank begins to rise at a potential of approximately 1.3 V and forms a “tail” at more positive potentials. Similar behavior can be observed for reduction at negative potentials (below −1 V). Since there is no formation of a distinct peak, it is evident that these oxidation and reduction processes are associated with the redox processes of water, which is inexhaustible. The oxidation of water is a highly complex process involving the production of various by-products, ultimately resulting in the formation of oxygen. Some of those by-products can undergo reduction at positive potentials, as indicated by a reduction peak around 1.4 V.

The voltammograms recorded with the perovskite-modified PIGE electrode (Figure 5—full lines) showed that all four perovskites exhibit electrochemical stability, demonstrating resistance to both self-oxidation and self-reduction when compared to the blank sample. This was concluded by recording several consecutive scans and obtaining very similar voltammograms. The electrochemical processes depicted in both positive and negative ends of the voltammograms are again associated with oxidation/reduction of the water. However, on perovskite-modified PIGE electrodes, these processes are more pronounced, requiring less potential energy, thus exhibiting good catalytic properties for all perovskites involved. When solely comparing the potential values (as current values cannot be precisely compared due to variations in the amount of perovskite on the electrode), PrMn_0.5_Co_0.5_O_3_ demonstrates the most favorable catalytical properties (Figure 5—red line). The reduction peak, occurring at a potential ranging from approximately −0.3 to −0.5 V, can also be observed as a small peak in the blank, and it is associated with the reduction of dissolved oxygen. This peak intensifies with all perovskites, but mostly in the perovskite with Co, once more demonstrating the superior catalytic properties of PrMn_0.5_Co_0.5_O_3_. Therefore, it can be concluded that all perovskites catalyze the redox processes of water and particularly the reduction of dissolved oxygen.

**Figure 6:**
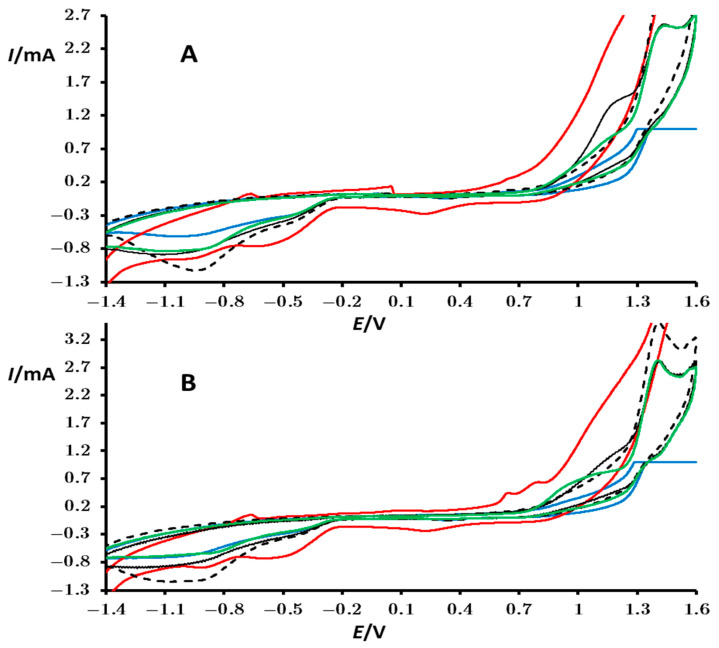
Cyclic voltammograms of a bare PIGE electrode (black dotted line) and PIGE modified with particles of PrMn_0.5_Ni_0.5_O_3_ (black), PrMn_0.5_Fe_0.5_O_3_ (green), PrMn_0.5_Cr_0.5_O_3_ (blue), and PrMn_0.5_Co_0.5_O_3_ (red) recorded in contact with a 0.5 mol/dm^3^ KNO_3_ and 0.1 mol/dm^3^ KOH. These measurements correspond to the first (**A**) and third (**B**) consecutive cycles. The potential was initially scanned from OCP in the positive direction. Additional conditions are specified in the Experimental Section.

The catalytic activity of the studied perovskite for oxidation of OH^−^ ions was studied in an aqueous solution of KNO_3_ (0.5 mol/dm^3^) containing 0.1 mol/dm^3^ KOH. Cyclic voltammograms for the first and third consecutive cycles are shown in Figure 6.

At around 1.4 V, an oxidation peak arising from the oxidation of hydroxide ions is evident in the voltammogram of the bare PIGE with KOH (0.1 mol/dm^3^) (Figure 6—black dotted line). This peak is absent in the blank without KOH (Figure 5—black dotted line), indicating that the observed signal is attributed to OH^−^ ions in the solution. This aligns with the literature, where oxidation of hydroxide ions typically occurs in the region of 1.3 to 1.6 V (vs. Ag/AgCl in 3 mol/dm^3^ KCl) [41,42]. At more positive potentials, the voltammogram displays signals associated with the redox processes of water, exhibiting a characteristic “tail”, similar to that observed without KOH (compare Figure 5 and Figure 6). This finding also aligns with the available literature data, where the potential for the oxygen evolution reaction (OER) from water oxidation is very close to that of the direct oxidation of OH^−^ ions; hence, a distinct peak cannot always be detected. However, voltammograms obtained with perovskite-modified PIGE electrodes show a noteworthy catalytic effect on the irreversible oxidation of OH^−^ ions by some of the studied perovskites, manifested by altered peaks within the oxidation potential range. Specifically, PrMn_0.5_Ni_0.5_O_3_ exhibits a peak at around 1.15 V in the first scan (Figure 6A—black line), while PrMn_0.5_Fe_0.5_O_3_ displays a peak at around 1 V in the third consecutive scan (Figure 6B—green line). The cyclic voltammogram of PrMn_0.5_Co_0.5_O_3_ is notably unique, showcasing rich electrochemistry in alkaline conditions, characterized by several peaks for oxidation and reduction and higher baseline current compared to other perovskites (Figure 6—red line). However, the formation of a well-defined and intense peak for oxidation is not observed, and the elevated baseline current may pose a challenge in detecting OH^−^. Therefore, the cyclic voltammograms suggest that PrMn_0.5_Fe_0.5_O_3_ and PrMn_0.5_Ni_0.5_O_3_ serve as effective catalysts for OH^−^ ions’ oxidation. The precise catalytic mechanism is somewhat complex and subject to debate in the literature, often attributed to the formation of hydroxide (M-OH bond) on the surface of the metals (in this case, perovskites) [43]. This behavior is consistent with the observation that consecutive cycling does not reproduce the same voltammograms (compare Figure 6A,B).

**Figure 7:**
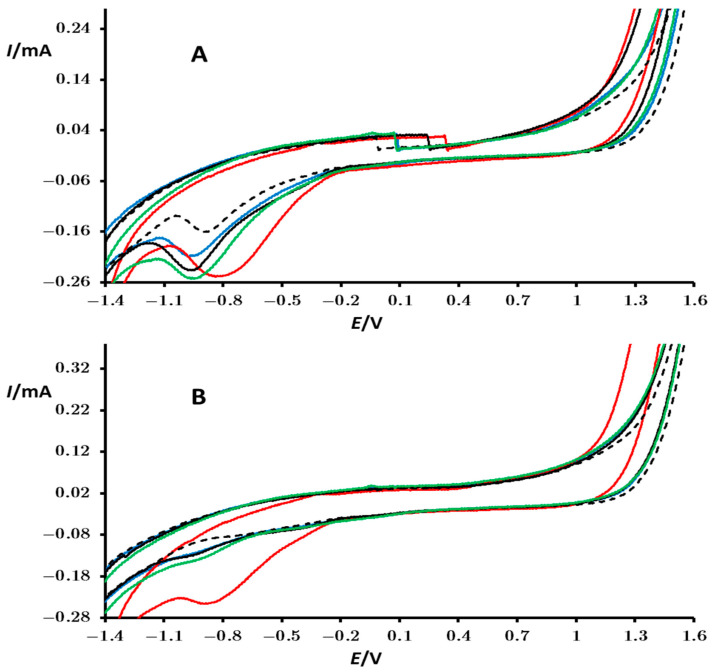
Cyclic voltammograms of a bare PIGE electrode (black dotted line) and of PIGE modified with particles of PrMn_0.5_Ni_0.5_O_3_ (black), PrMn_0.5_Fe_0.5_O_3_ (green), PrMn_0.5_Cr_0.5_O_3_ (blue), and PrMn_0.5_Co_0.5_O_3_ (red) recorded in contact with a 0.1 mol/dm^3^ phosphate buffer. These measurements correspond to the first (**A**) and third (**B**) consecutive cycles. The potential was initially scanned from OCP in the positive direction. Additional conditions are specified in the Experimental Section.

The electrocatalytic activity of perovskites toward H_2_O_2_ oxidation/reduction was investigated in a phosphate buffer (pH = 7.35). To assess the influence of H_2_O_2_ in the voltammograms, cyclic voltammograms for all perovskites were recorded under identical conditions, but without H_2_O_2_ (Figure 7).

Similar to voltammograms in KNO_3_, cyclic voltammograms of all perovskites in phosphate buffer exhibit no peaks associated with the electrochemistry of the perovskites, indicating their stability in this potential region (Figure 7). However, there are discrepancies between the peak for reduction of oxygen and the “tail” for oxidation of water under both conditions. Specifically, compared to KNO_3_, in phosphate buffer, the peak potential for reduction of oxygen is 300 mV lower, and a reduction peak is absent at 1.4 V (compare black dotted lines in Figure 5 and Figure 7). These distinct characteristics under both conditions underscore the complexity of these processes.

During the initial scan, all perovskites exhibit catalytic activity (with increased peak current) for the reduction of oxygen at potentials ranging from −0.8 to −1 V, with the lowest negative potential, and hence the highest catalytic activity observed for PrMn_0.5_Co_0.5_O_3_ (Figure 7A—red line). This same perovskite also demonstrates the highest catalytic activity for the oxidation of water. Notably, for dissolved oxygen, the reduction peak is significantly diminished in subsequent cycling for all perovskites, except for PrMn_0.5_Co_0.5_O_3_ (Figure 7B). This behavior indicates that the catalyzed oxidation of water at PrMn_0.5_Co_0.5_O_3_ efficiently regenerates new oxygen on the surface of the electrode, a phenomenon not observed with other perovskites.

**Figure 8:**
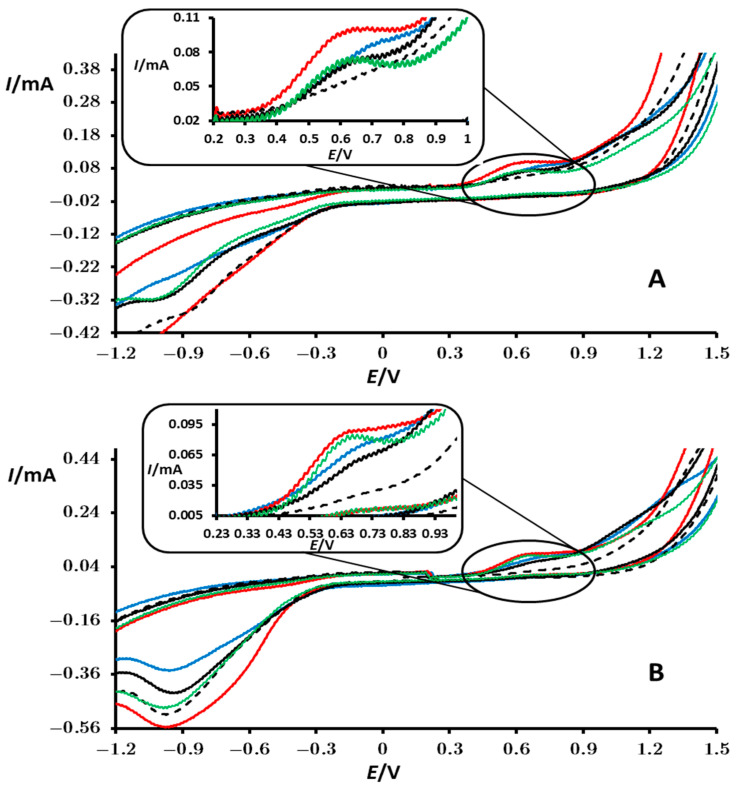
Cyclic voltammograms of a bare PIGE electrode (black dotted line) and of PIGE modified with particles of PrMn_0.5_Ni_0.5_O_3_ (black), PrMn_0.5_Fe_0.5_O_3_ (green), PrMn_0.5_Cr_0.5_O_3_ (blue), and PrMn_0.5_Co_0.5_O_3_ (red) recorded in contact with a 0.1 mol/dm^3^ phosphate buffer and 10^−2^ mol/dm^3^ H_2_O_2_. These measurements correspond to the first (**A**) and third (**B**) consecutive cycles. The potential was initially scanned from OCP in the positive direction. Additional conditions are specified in the Experimental Section.

The addition of H_2_O_2_ to phosphate buffer on a bare PIGE results in a higher current in cyclic voltammograms at both ends of the voltammogram. The oxidation of H_2_O_2_ is observed at very positive potentials (above 1.1 V), while reduction begins around −0.4 V (Figure 8—black dotted lines). These processes intermingle with the oxidation/reduction of water and the reduction of oxygen, often manifesting as a “tail”, particularly for the oxidation process.

The voltammograms recorded for the redox processes of H_2_O_2_ in phosphate buffer on the perovskite-modified PIGE electrode exhibit a similar shape compared to those for the bare electrode. However, the appearance of an irreversible oxidation peak spanning the range of 0.4–0.8 V with a peak potential of 0.6 V (inset in Figure 8) is of crucial importance. This peak remains relatively stable after consecutive cycles (compare Figure 8A,B), observed only in the presence of H_2_O_2_ when a perovskite-modified PIGE electrode is used, and has the highest peak current in the case of iron- and cobalt-containing perovskites (Figure 8, green and red lines, respectively). As for the peak potentials, it seems that they also vary slightly and are lower for the Co and Fe perovskites compared to those for Ni and Cr perovskites. This confirms that the Co- and Fe-containing perovskites are better catalysts for H_2_O_2_ oxidation than those containing Ni and Cr. The precise elucidation of the mechanism behind the oxidation of H_2_O_2_ and linking the peak to a specific electrode reaction is not the primary focus of this article. However, similar peaks have been observed in several articles in the literature concerning other perovskite-modified electrodes [44,45].

## 4. Conclusions

The perovskites with the general formula PrMn_0.5_M_0.5_O_3_ (M = Cr, Fe, Co, Ni) were successfully obtained by the solution combustion method using glycine as fuel, and PrMn_0.5_Fe_0.5_O_3_ was also synthesized by the sol–gel combustion method with citric acid as fuel. The XRPD patterns showed that the perovskites are pure and isostructural to each other within the series. The diffractograms of PrMn_0.5_Fe_0.5_O_3_ obtained by the two different methods are identical, indicating that both methods can be used for perovskite synthesis.

The solution combustion method of synthesis of double perovskites usually results in the formation of an orthorhombic phase. This was confirmed by the determination of the lattice cell parameters in the *Pnma* space group and the calculation of several crystallochemical parameters indicating the structure distortion. The obtained perovskites are only slightly distorted, and the most stable is PrMn_0.5_Ni_0.5_O_3_.

The SEM images revealed the porous morphology of the obtained perovskites as expected when solution combustion synthesis is applied. The Pr:Mn:M ratio of the atomic percentages obtained by EDX analysis, in all cases, is approximately 2:1:1.

Investigations on the electrocatalytic properties of the perovskites of PrMn_0.5_M_0.5_O_3_ (M = Cr, Fe, Co, and Ni) using cyclic voltammetry demonstrated their catalytic activity in processes of OH^−^ oxidation, H_2_O_2_ oxidation in a phosphate buffer, and partial catalysis of water oxidation and reduction and reduction of dissolved oxygen. The most pronounced electrocatalytic activity toward OH^−^ oxidation exhibits PrMn_0.5_Fe_0.5_O_3_. In the case of H_2_O_2_ oxidation in a phosphate buffer, both PrMn_0.5_Fe_0.5_O_3_ and PrMn_0.5_Co_0.5_O_3_ exhibit significant electrocatalytic activity with a distinct oxidation peak with a peak potential of 0.6 V.

## Figures and Tables

**Table 1:** Calculated unit cell parameters (*a*, *b*, *c*), unit cell volume (V), tolerance factor (*t*), spontaneous strain (*s*), pseudocubic parameter (*a*_p_), unit cell distortion (*d*), and orthorhombic distortion (*dist*_orth_) for PrMn_0.5_M_0.5_O_3_ perovskites.

Parameter	PrMn_0.5_Cr_0.5_O_3_	PrMn_0.5_Fe_0.5_O_3_	PrMn_0.5_Co_0.5_O_3_	PrMn_0.5_Ni_0.5_O_3_
*a*/Å	5.48332(15)	5.50542(10)	5.44150(7)	5.44720(5)
*b*/Å	7.71590(16)	7.76003(16)	7.71534(14)	7.70407(9)
*c*/Å	5.45981(13)	5.47908(9)	5.41677(56)	5.44336(47)
*β*/°	90	90	90	90
*V*/Å^3^	230.998	234.078	227.412	228.431
*t*	0.8983	0.8917	0.9141	0.9016
*s*	0.004297	0.004796	0.004555	0.000716
*a*_p_/Å	3.86530	3.88241	3.845209	3.850927
*d*	0.04893	0.04026	0.08698	0.001269
(*b/*√2)/Å	5.455965	5.48717	5.455569	5.4476
*dist*_orth_	0.003831	0.003475	0.005108	0.000617

**Table 2:** EDX analysis of Pr_2_MnMO_6_ (M = Ni, Co, Cr, Fe).

PrMn_0.5_Cr_0.5_O_3_		PrMn_0._5Fe0_.5_O_3_		PrMn_0.5_Co_0.5_O_3_		PrMn_0.5_Ni_0.5_O_3_	
Element	Atomic %	Element	Atomic %	Element	Atomic %	Element	Atomic %
Mn	3.09	Mn	3.74	Mn	6.85	Mn	5.07
Cr	2.50	Fe	3.51	Co	6.23	Ni	5.89
Pr	5.43	Pr	6.51	Pr	10.72	Pr	10.13

## Data Availability

The datasets presented in this article are not readily available because the data are part of an ongoing study. Requests to access the datasets should be directed to besartaceliku@hotmail.com.

## Supplementary Materials

The following supporting information can be downloaded at: https://www.mdpi.com/article/10.3390/ma18030717/s1, Figure S1: Schematic representation of an electrochemical cell showing reference, counter and working electrodes (A). An image of the modified PIGE electrode (B); Figure S2: EDX data for the pervoskites; Tables S1–S4. Amounts used for each reactive.
