# Supplementary material for: Synthesis, Characterization, and Electrocatalytic Properties of PrMn0.5M0.5O3 (M = Cr, Fe, Co, Ni) Perovskites
Source: Materials (Basel). 2025 Feb 6;18(3):717. doi: 10.3390/ma18030717 (PMC11820077; doi:10.3390/ma18030717)
Supplement: Supplementary file 1 [file materials-18-00717-s001.zip › materials-3449180-supplementary.pdf]

Tables S1-S4. Amounts used for each reactive

$PrMn_{0.5}Cr_{0.5}O_3$	
$Pr(NO_3)_3 \cdot 5H_2O$	1,718g
$Mn(NO_3)_2 \cdot 4H_2O$	0.517g
$Cr(NO_3)_3 \cdot 9H_2O$	0.824g
$C_2H_5NO_2$	0.938g

$PrMn_{0.5}Fe_{0.5}O_3$	
$Pr(NO_3)_3 \cdot 5H_2O$	1,705g
$Mn(NO_3)_2 \cdot 4H_2O$	0,513g
$Fe(NO_3)_3 \cdot 9H_2O$	0,826g
$C_2H_5NO_2$	0,937g

$PrMn_{0.5}Co_{0.5}O_3$	
$Pr(NO_3)_3 \cdot 5H_2O$	1,696g
$Mn(NO_3)_2 \cdot 4H_2O$	0,510g
$Co(NO_3)_2 \cdot 6H_2O$	0,592g
$C_2H_5NO_2$	0,848g

$PrMn_{0.5}Ni_{0.5}O_3$	
$Pr(NO_3)_3 \cdot 5H_2O$	1,696g
$Mn(NO_3)_2 \cdot 4H_2O$	0,510g
$Ni(NO_3)_2 \cdot 6H_2O$	0,591g
$C_2H_5NO_2$	0.848g

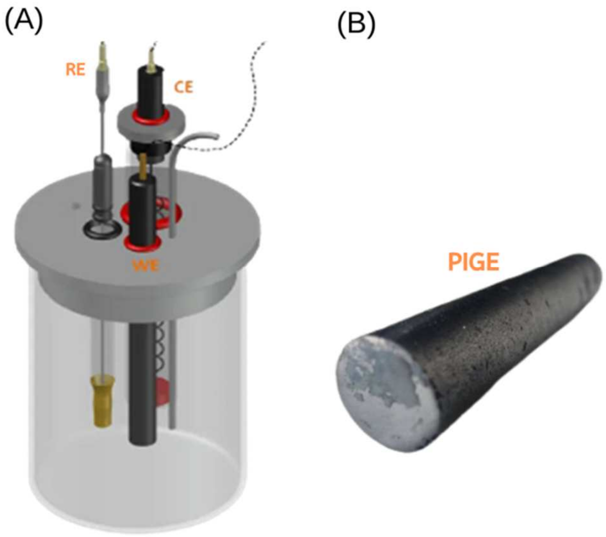

Figure S1. Schematic representation of an electrochemical cell showing reference, counter and working electrodes (A). An image of the modified PIGE electrode (B).

$PrMn_{0,5}Cr_{0,5}O_3$

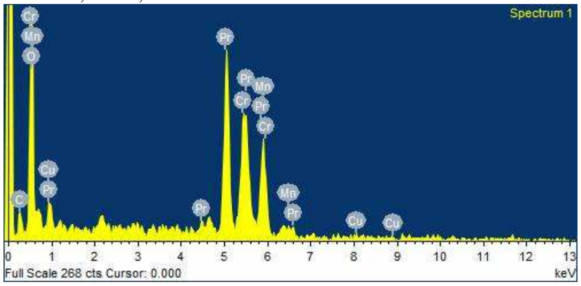

$PrMn_{0,5}Fe_{0,5}O_3$

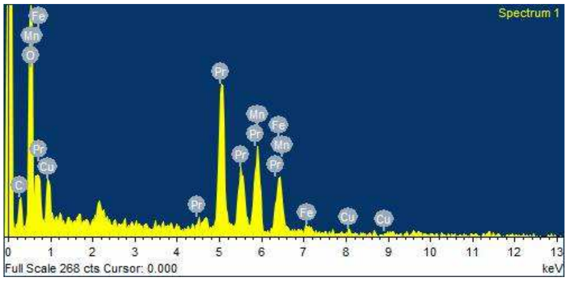

$PrMn_{0,5}Co_{0,5}O_3$

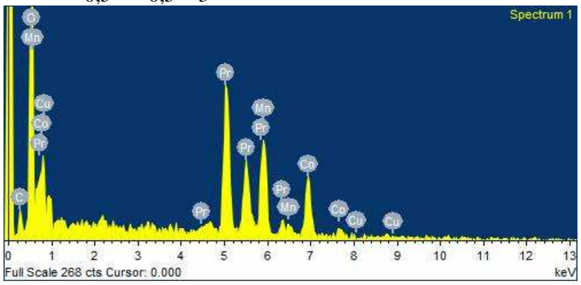

$PrMn_{0,5}Ni_{0,5}O_3$

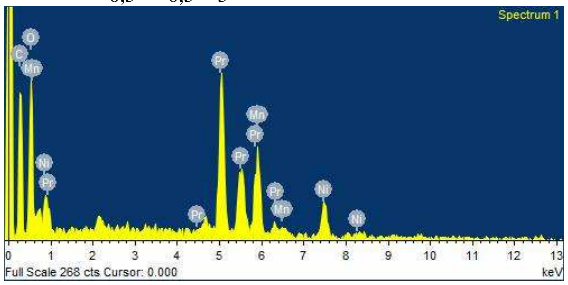

Figure S2. EDX data for the pervoskites
